# Genetic Analysis Workshop 13: Analysis of Longitudinal Family Data for Complex Diseases and Related Risk Factors

**DOI:** 10.1186/1471-2156-4-S1-S1

**Published:** 2003-12-31

**Authors:** Laura Almasy, Christopher I Amos, Joan E Bailey-Wilson, Rita M Cantor, Cashell E Jaquish, Maria Martinez, Rosalind J Neuman, Jane M Olson, Lyle J Palmer, Stephen S Rich, M Anne Spence, Jean W MacCluer

**Affiliations:** 1 Department of Genetics, Southwest Foundation for Biomedical Research, San Antonio, Texas, 78245, USA; 2 Department of Epidemiology, University of Texas MD Anderson Cancer Center, Houston, Texas, 77025, USA; 3 National Human Genome Research Institute, National Institutes of Health, Baltimore, Maryland, 21224, USA; 4 Department of Human Genetics, University of California Los Angeles School of Medicine, Los Angeles, California, 90095, USA; 5 National Heart Lung and Blood Institute, National Institutes of Health, Bethesda, Maryland, 20892, USA; 6 INSERM, Hôpital Saint Louis, Evry Cedex, 91034, France; 7 Department of Psychiatry, Washington University School of Medicine, St. Louis, Missouri, 63110, USA; 8 Department of Biostatistics and Epidemiology, Case Western Reserve University, Cleveland, Ohio, 44109, USA; 9 Genetic Epidemiology Group, Western Australia Institute for Medical Research, Perth, WA 6000, Australia; 10 Department of Public Health Sciences, Wake Forest University School of Medicine, Winston-Salem, North Carolina, 27517, USA; 11 Department of Pediatrics, University of California Irvine Medical Center, Orange, California, 92868, USA

## Preface

This supplement to BMC Genetics contains the proceedings of Genetic Analysis Workshop 13 (GAW13), which was held November 11–14, 2002, at the Marriott hotel in New Orleans, Louisiana. The Genetic Analysis Workshops, which began in 1982 and are currently held biannually, provide a venue for the development and testing of statistical genetic methods. For each GAW, one or more data sets are made available to potential participants and GAW attendees must submit an analysis of one of these data sets, or be the providers of a data set or otherwise involved in workshop organization. The workshop itself is then a head-to-head comparison of different ways of analyzing the same data. New analytical methods are introduced and the performance of established methods is compared.

GAW13 focused on analytical issues relating to localization of genes influencing common, complex diseases and their risk factors, with an emphasis on use of longitudinal data. For the first time in a GAW, longitudinal family data, covering a 40-year time period, was made available to workshop participants. As has been the case in recent GAWs, both simulated and actual human data were available. New this year, however, was a close correspondence between these data sets, with the simulated data mirroring the family structure, trait data, and longitudinal sampling scheme of the real data set.

The Framingham Heart Study data provided for GAW13 included longitudinal observations from two cohorts. The original Framingham cohort (Cohort 1), was first examined in 1948 and has been examined every 2 years thereafter. Cohort 2, composed primarily of offspring of the original cohort and the spouses of these offspring, was examined first in 1971 and has been examined approximately every 4 years. The data provided for GAW13 are a subset of these two cohorts and include 2885 individuals in 300 pedigrees. Both diagnostic phenotypes, such as hypertension, and quantitative risk factors, such as HDL cholesterol levels, weight, and systolic blood pressure were available. Genotypes were provided for microsatellite markers representing a whole genome scan at an average 10-cM density. Marker maps and estimated marker allele frequencies were also supplied.

A second data set was simulated, based as closely as possible on the Framingham Heart Study data made available to GAW13. While the exact details of the pedigrees were modified, the overall pedigree size and structure in the simulated data was based on that of the Framingham Heart Study data. Similarly, phenotypes were simulated to have the same distribution as those in the real data and the observed correlations between some phenotypes were incorporated into the trait model. Genotype data were also closely matched to the actual data, particularly in regard to marker distribution and information content. While participants had the option of using the complete simulated data set, missing data patterns were also generated based on observations from the Framingham Heart Study data. Those who analyzed the simulated data had the choice of obtaining the simulation model prior to their analyses and were asked to indicate whether their analyses were done with or without this knowledge.

In spring of 2002, the availability of the GAW13 data was announced by e-mail to the over 1700 individuals on the GAW mailing list. A total of 97 groups requested GAW13 data. The Framingham data were requested by 75 groups and the simulated data by 90 groups, with 67 groups requesting both data sets. In the summer of 2002, 117 contributions were received describing analyses of the two data sets. Of these, 81 utilized the Framingham Heart Study data and 36 analyzed the simulated data. A book, or CD, containing these contributions plus papers describing the data sets was distributed to workshop participants.

A total of 241 individuals from 13 countries attended GAW13. Attendees included investigators from five continents – Asia, Australia, Europe, and North and South America. As with GAW12, participants were organized into 11 presentation groups and a co-author with past GAW experience was asked to lead discussion in each presentation group. Groups ranged in size from 6 to 14 papers with common themes. Some groups collected papers exploring similar methodological issues, such as methods for longitudinal analysis or derivation of new phenotypes from the data, whereas others were related through a common focus on particular phenotypes, such as analyses of blood pressure and hypertension phenotypes or analyses of tobacco and alcohol use. Given the similarities in the Framingham Heart Study and simulated data sets, presentation groups were assigned without regard to the data set analyzed. Each group met individually during the workshop and in many groups members communicated beforehand to begin comparing and contrasting the approaches taken and the results obtained by group members. At GAW13, many groups used part of their group meeting time for individual presentations by each group member, giving investigators an opportunity to present their work. Although mainly attended by group members, group meetings were open to all GAW13 participants. From this process, each group developed an oral presentation, summarizing and synthesizing the work of the individual papers, which was delivered to the overall workshop during general sessions. Individual contributions were also presented in the form of 52 posters displayed during four poster sessions.

The manuscripts included here are a subset of those presented at GAW13. All of these papers have been reviewed for scientific merit. The proceedings begin with two papers describing the two data sets, followed by 103 individual GAW13 contributions organized by presentation group and alphabetically by first author within each group. Because the data set analyzed was not considered in assignment of group membership, papers reporting analyses of simulated data and Framingham Heart Study data are intermingled. New to GAW this year, the proceedings occupy two volumes. In addition to the individual contributions in this volume, each presentation group has a summary in a forthcoming supplement to the journal *Genetic Epidemiology *in which the present manuscripts are compared and contrasted and larger themes and conclusions are explored. Results of GAW13 analyses provided novel insights into the etiology of cardiovascular disease and some of its risk factors. In addition, many new methods were developed, explored, and applied for the analysis of genetic data from longitudinal studies, an area of research that has been underdeveloped to date.

